# Circular RNA erythrocyte membrane protein band 4.1 assuages ultraviolet irradiation-induced apoptosis of lens epithelial cells by stimulating 5’-bisphosphate nucleotidase 1 in a miR-24-3p-dependent manner

**DOI:** 10.1080/21655979.2021.1990196

**Published:** 2021-10-28

**Authors:** Cuiyun Zhou, Xiaoqiong Huang, Xia Li, Yan Xiong

**Affiliations:** Department of Ophthalmology, Jingmen First People’s Hospital, Jingmen, Hubei, China

**Keywords:** ARC, UV, circ_EPB41, miR-24-3p, bpnt1

## Abstract

Apoptosis of lens epithelial cells contributed to the formation of age-related cataract (ARC), and previous data revealed that circular RNA (circRNA) was responsible for the underneath mechanism. The study was organized to explore the role of circular RNA erythrocyte membrane protein band 4.1 (circ_EPB41) in ultraviolet (UV) irradiation-induced apoptosis of lens epithelial cells. SRA01/04 cells were irradiated with UV to mimic the ARC cell model. The RNA levels of circ_EPB41, microRNA-24-3p (miR-24-3p), and 3ʹ(2ʹ), 5ʹ-bisphosphate nucleotidase 1 (BPNT1) were detected by quantitative real-time polymerase chain reaction. Protein expression was checked by western blot. 5-Ethynyl-29-deoxyuridine, 3-(4,5-Dimethylthazol-2-yl)-2,5-diphenyltetrazolium bromide and DNA content quantitation assays were performed to investigate cell proliferation. Flow cytometry was conducted to analyze cell apoptosis. Dual-luciferase reporter assay was implemented to confirm the interaction among circ_EPB41, miR-24-3p, and BPNT1. Our data showed that circ_EPB41 and BPNT1 expression were downregulated in ARC tissues and UV-irradiated SRA01/04 cells as compared with normal anterior lens capsules and untreated SRA01/04 cells. Circ_EPB41 overexpression ameliorated the effects of UV irradiation on the proliferation and apoptosis of SRA01/04 cells. Besides, miR-24-3p, a target miRNA of circ_EPB41, attenuated circ_EPB41 introduction-mediated proliferation, and apoptosis of UV-irradiated SRA01/04 cells. MiR-24-3p regulated UV irradiation-induced effects by targeting BPNT1. Importantly, it was found that circ_EPB41 stimulated BPNT1 production by miR-24-3p. Taken together, the enforced expression of circ_EPB41 ameliorated UV irradiation-induced apoptosis of lens epithelial cells by miR-24-3p/BPNT1 pathway, providing us with a potential target for the therapy of UV-caused ARC.

## Introduction

Cataract is the most common eye disease and represents more than 40% of all causes of blindness [1]. Age-related cataract (ARC) is featured by the opacity of the lens in the eye. Considered data have confirmed a notable increase in lens epithelial cell apoptosis during ARC [2], which suggests that lens epithelial cell apoptosis may be a common cell process for ARC. Radiation of ultraviolet (UV), especially those with a wavelength longer than 290 nm, is considered to be the direct pathogenic factor for ARC [3]. Thus, the investigation focusing on the apoptosis of lens epithelial cells (LECs) induced by UV radiation may shed new light in developing the reliable target for the therapy of ARC.

Regarded as a brand-new star molecule, circRNA is transcribed from linear RNA [4]. CircRNA is an endogenous conserved transcript that forms a covalently closed-cyclic structure through head-to-tail splicing, without protein-coding ability [5]. In contrast with linear RNA, carrying ≥7 nucleotides required for exoribonuclease, circRNA is able to resist RNase or RNA exonucleases-induced degradation [6]. On that account, circRNA is commonly employed as a diagnostic or therapeutic marker for various diseases. Large-scale studies indicate that circRNA can sink microRNAs (miRNAs) or interact with RAN-binding proteins to regulate the function processed by the miRNAs or target gene expression [7]. Multiple human diseases see dysregulated expression of circRNA, which is associated with the physiology and pathology of different diseases [8]. Recent studies have also identified the regulatory role of circRNA in age-related diseases, such as cardiovascular disease [9], Alzheimer’s disease [10], and cataract [11]. Circular RNA erythrocyte membrane protein band 4.1 (circ_EPB41), also termed as circ_0011173, has been discovered to be downregulated in ARC tissues as compared with transparent lens through hierarchical clustering analysis [12]. However, the underlying mechanism has not been reported, especially with respect to the function of circ_EPB41 in UV irradiation-induced apoptosis of LECs.

The miRNA response elements (MREs) of the circRNAs make them able to interact with miRNAs [13]. As a class of small single-stranded RNAs, miRNAs inhibit genes or noncoding RNAs through regulating their translation or degradation. It has been estimated almost one-third of the protein-coding genes are responsible for the function of miRNAs in diseases [14]. Accumulating evidences demonstrated the involvement of miRNAs in cataract. For example, miR-22-3p prevented proliferation and migration of Transforming Growth Factor-β2 (TGF-β2)-stimulated LECs by binding to histone deacetylase 6 (HDAC6) to induce α-tubulin acetylation [15]. Xiu *et al*. reported cataract occurrence involved the upregulation of miR-34a, which might be associated with the regulation of sirtuin 1 [16]. In particular, previous evidence has confirmed the upregulation of miR-24-3p in cataractous lenses in comparison with transparent lenses [17]. However, few data about the role of miR-24-3p in ARC formation were reported.

Based on the ceRNA hypothesis, the study analyzed whether circ_EPB41 was able to interact with miR-24-3p. As a result, we found miR-24-3p carried the binding sites of circ_EPB41. Also, it was testified that miR-24-3p was capable of targeting 5ʹ-bisphosphate nucleotidase 1 (BPNT1). Thus, we hypothesized that circ_EPB41 regulated ARC formation by miR-24-3p/BPNT1 axis. Given the absence of data involving circ_EPB41/miR-24-3p/BPNT1 axis in ARC occurrence, we designed the study to explore whether UV irradiation-induced apoptosis of LECs involved the regulatory route, so as to provide a potential target for the therapy of UV-caused ARC.

## Materials and methods

### Sample collection

The Ethics Committee of Jingmen First People’s Hospital approved the study. Nineteen anterior lens capsules were collected from ARC patients (age range 55–75 years) who underwent ARC surgery at Jingmen First People’s Hospital. Thirteen transparent anterior lens capsules from postmortem eyes were used as normal controls. These anterior lens capsules were frozen in liquid nitrogen. All participants were free of ocular diseases, and signed written informed consent.

## Cell culture and UV irradiation treatment

Human LECs (SRA01/04; Bluefbio, Shanghai, China) were maintained in Dulbecco’s modified Eagle’s medium (DMEM; Biosun, Shanghai, China) added with 10% fetal bovine serum (FBS; Biosun) and 1% penicillin/streptomycin (Phygene, Fuzhou, China) at 37°C in an atmosphere of 5% CO_2_. To simulate the ARC cell model *in vitro*, SRA01/04 cells cultured in 0.5 mL phosphate buffer solution (PBS; Biosun) were irradiated without a plastic dish lid using 15-watt UVB lamps (Spectroline, Westbury, NY, USA) at an intensity of 360 μW/cm^2^ for 30 min. Then, PBS was removed and fresh DMEM was added to the culture plates.

## Cell transfection

According to the published method [18], plasmids and oligonucleotides were transfected into the SRA01/04 cells at ~70% confluence using transfection reagent (Thermo Fisher, Waltham, MA, USA). The complete sequence of circ_EPB41 amplified by polymerase chain reaction (PCR) was inserted into pCD5-ciR (Vector; Geneseed, Guangzhou, China) to build the plasmid overexpressing circ_EPB41 (circ_EPB41). Songon Biotech (Shanghai, China) provided the oligonucleotides, including the mimics and inhibitors of miR-24-3p (miR-24-3p 5ʹ-UGGCUCAGUUCAGCAGGAACAG-3ʹ and anti-miR-24-3p 5ʹ-CUGUUCCUGCUGAACUGAGCCA-3ʹ), the small interfering RNAs against BPNT1 (si-BPNT1 5ʹ-GGGTATTGTGGAGAAGACCTGTGCA-3ʹ), and circ_EPB41 (si-circ_EPB41 5ʹ-AGCATGGAAGCAAGAGCAGTA-3ʹ), and respective controls (miR-NC, anti-miR-NC, and si-NC).

## Quantitative real-time PCR (qRT-PCR)

TsingZol (Tsingke, Shanghai, China) was utilized to isolate RNA, and RNA quality was analyzed using NanoDrop-1000 instrument (Thermo Fisher). The RNA was reversely transcribed to complementary DNA (cDNA) by using high-capacity cDNA synthesis kits purchased from Ribobio Co., Ltd. (Guangzhou, China) or TaKaRa (Dalian, China) following the guidebooks. For analysis of gene expression, qRT-PCR reaction was performed on a Bio-Rad 96-well qRT-PCR machine (Hercules, CA, USA) after the cDNA was mixed with primers and T5 Fast qPCR Mix (Tsingke). At last, gene expression was calculated by the 2^−∆∆Ct^ method [19] with the small nuclear RNA U6 (U6) and glyceraldehyde 3-phosphate dehydrogenase (GAPDH) as controls. Primer sequences were listed in Table 1.

**Table 1.** Primers sequences used for qRT-PCR

Name		Sequences (5ʹ-3ʹ)
circ_EPB41	Forward	CCTAGATGCCTCTGCTAAA
	Reverse	GCTCGGTAACTGGGAAGT
EPB41	Forward Reverse	TGCGTTTAGTCAGTCAGCCA TTGTCATGATGTTGCGGTGG
BPNT1	Forward	CACTGTGTTGATGCGGTTGG
	Reverse	GTGCCAATCGGTCAGCTTTG
miR-24-3p	Forward	GATCCTGGCTCAGTTCAGCAG
	Reverse	AGTGCAGGGTCCGAGGTATT
GAPDH	Forward	CAAATTCCATGGCACCGTCA
	Reverse	GACTCCACGACGTACTCAGC
U6	Forward	CTTCGGCAGCACATATACT
	Reverse	AAAATATGGAACGCTTCACG

## Stability analysis of circRNA

SRA01/04 cells were co-cultured with Actinomycin D (Amresco, Solon, OH, USA) at a concentration of 2 μg/mL for 0, 8, 16, and 24 h based on the reported method [20], followed by RNA isolation. Circ_EPB41 expression was checked by qRT-PCR with the mRNA of EPB41 as a reference.

## Subcellular fractionation location assay

A total of 10^7^ SRA01/04 cells were collected and then placed in a microfuge tube. Then, nucleocytoplasmic separation was carried out with a PARIS™ Kit (Thermo Fisher) as instructed [21]. After isolation of RNA from nuclear pellet and cytoplasmic sections, circ_EPB41 was quantified by qRT-PCR with a high-capacity cDNA synthesis kit (Thermo Fisher). GAPDH and U6 acted as references.

## 5-Ethynyl-29-deoxyuridine (Edu) assay

SRA01/04 cells were grown in 12-well plates (1 × 10^5^ cell per hole) and irradiated with UV or transfected with circ_EPB41, vector, miR-24-3p, miR-NC, anti-miR-24-3p, anti-miR-NC, si-BPNT1, or si-NC. Post-transfection of 48 h, the cells were mixed with an Edu-labeled medium and then added to 96-well plates. Then, cell proliferation was confirmed by analyzing the number of Edu-positive cells with an Edu staining kit (Ribobio) as per the guidebook. The staining results were captured by using a fluorescence microscope (Olympus, Tokyo, Japan).

## 3-(4,5)-dimethylthiahiazo (-z-y1)-3,5-di-phenytetrazoliumromide (MTT) assay

The SRA01/04 cells cultured in 96-well plates (5000 cells per well) were treated with UV irradiation, plasmids, or oligonucleotides, followed by culture for 0, 1, 2 and 3 days. The following procedures were conducted as shown previously [22]. In brief, the cells were mixed with MTT solution (Abcam, Cambridge, MA, USA) for 4 h. Then, dimethyl sulfoxide (Seebio Biotech, Shanghai, China) was used to dissolve the forming formazan. Eventually, the output of optical density (OD) at 570 nm was read by a microplate reader (Thermo Fisher).

## Western blot

The assay used to detect protein expression was performed as instructed [23]. Proteins were extracted using a protein extraction kit (Phygene) according to the instructions of the manufacturer. After the denaturation at 95°C, the protein samples were equivalently added to SurePAGE gels (GenScript, Nanjing, China) and wet-transferred onto nitrocellulose membranes (GenScript) by using an eBlot™ L1 protein transfer system (GenScript). Then, skimmed milk was used to block the aspecific signals. The membranes were incubated with primary antibodies specific to anti-proliferating cell nuclear antigen (PCNA), B-cell lymphoma-2 (Bcl-2), BCL2-associated x protein (Bax), BPNT1, and GAPDH at a dilution of 1:1000. The membranes were incubated with goat anti-rabbit IgG, and then exposed to an X-ray film to develop protein blots. GAPDH acted as a loading control. The antibodies against BPNT1 and GAPDH were purchased from Otwo Biotech (Shenzhen, China), and other antibodies were provided by Abcam Biotech Corporation. GAPDH was employed for the normalization of protein expression.

## Flow cytometry analysis

At 48 h after various treatments, the cells were digested, collected, and washed using PBS (Biosun). Apoptosis detection kit (Solarbio, Beijing, China) was then used to quantify the apoptotic rate of the cells as per the guidebook. In brief, the cells were incubated with Binding Buffer for 10 min. Subsequently, Annexin V-FITC and propidium iodide (PI) staining solutions were used to incubate the cells in the dark. At last, apoptotic cells were quantified by a flow cytometer (Thermo Fisher).

## Cell-cycle analysis by flow cytometry

The assay regarding cell cycle was performed using DNA content quantitation assay kit (Solarbio) following the instruction of manufacture. In brief, the cells were washed and centrifuged at 1500 rpm for 5 min. After being fixed using ethanol, these cells were incubated with RNase A and PI, respectively. Finally, samples were analyzed using a flow cytometer (Thermo Fisher).

## Dual-luciferase reporter assay

Online databases Circbank (http://www.circbank.cn/index.html) and Starbase (http://starbase.sysu.edu.cn/agoClipRNA.php?source=mRNA) were used to predict the binding sites of circ_EPB41 and BPNT1 for miR-24-3p. Based on the complementary sites, the sequences of circ_EPB41 and BPNT1 carrying the binding sites of miR-24-3p were synthesized by Generay (Shanghai, China) to build the wild-type (WT) plasmids including circ_EPB41-WT and BPNT1-WT with pmirGLO vector (Miaoling, Wuhan, China), which carried both *Renilla* and *firefly* luciferase reporter genes. In the same manner, the complementary sites of circ_EPB41 and BPNT1 with miR-24-3p were mutated by GenScript Biotech Corporation to build mutant (MUT) plasmids (circ_EPB41-MUT and BPNT1-MUT). The above plasmids were co-transfected into SRA01/04 cells with miR-24-3p mimics or mimics control using TurboFect reagent (Thermo Fisher) as per the guidebook. The cells went through 48-hour incubation and Duo-Lite^TM^ Luciferase Assay kit (Vazyme, Jiangsu, China) was used to identify the binding intensity inferring to the guidebook.

## Statistical analysis

All experimental data were obtained from three independent duplicate tests, and described as means ± standard deviations. The significant differences in Spearman correlation analysis were analyzed by Spearman’s correlation test. Wilcoxon rank-sum test and two-tailed Student’s *t*-tests were used to compare the significant differences between the two groups, and one-way analysis of variance was used among three or more groups. All data analysis was performed on GraphPad Prism software. *P* value <0.05 was considered as statistical significance.

## Results

In the present study, we explored the role of circ_EPB41 in the apoptosis of LECs induced by UV radiation and the underlying mechanism. Our results showed that circ_EPB41 was downregulated in ARC tissues and UV-irradiated SRA01/04 cells. Increasing circ_EPB41 expression ameliorated the effects of UV irradiation on the proliferation and apoptosis of SRA01/04 cells. Besides, it was found that circ_EPB41 regulated BPNT1 production through miR-24-3p. Overall, our data demonstrated that enforced expression of circ_EPB41 assuaged UV irradiation-caused apoptosis of lens epithelial cells by miR-24-3p/BPNT1 axis, providing us with a potential target for the therapy of UV-caused ARC.

### Circ_EPB41 expression was downregulated in ARC tissues and UV-irradiated SRA01/04 cells

**Figure 1.**
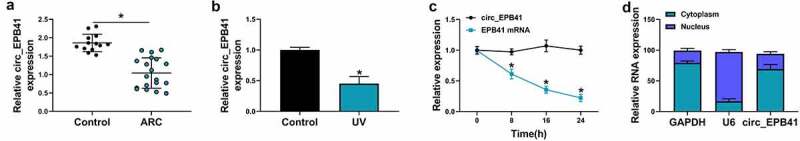
The expression of circ_EPB41 in ARC tissues and in SRA01/04 cells treated with UV irradiation. (a and b) Circ_EPB41 level was detected by qRT-PCR in anterior lens capsules of ARC (N = 19), transparent anterior lens capsules (N = 13), SRA01/04 cells treated with UV irradiation and untreated SRA01/04 cells. (c) The stability of circ_EPB41 was identified by Actinomycin D treatment assay in SRA01/04 cells. (d) Subcellular fractionation location assay was performed to confirm the location of circ_EPB41 in SRA01/04 cells. *P < 0.05

Circ_EPB41 expression was firstly detected in ARC tissues. As shown in Figure 1a, circ_EPB41 expression was lower in anterior lens capsules of ARC compared with transparent anterior lens capsules. Also, we found that the SRA01/04 cells treated with UV irradiation displayed a weak expression of circ_EPB41 in comparison with untreated SRA01/04 cells (Figure 1b). Actinomycin D treatment assay showed the transcript half-life of circ_EPB41 exceeded 24 h, although that of linear EPB41 was about 8 h (Figure 1c), suggesting the high stability of circ_EPB41. Further data exhibited that circ_EPB41 was mainly located in the cytoplasm of SRA01/04 cells (Figure 1d). The above data indicated that the mechanism behind the ARC formation might be associated with circ_EPB41.

## Circ_EPB41 overexpression ameliorated UV irradiation-induced apoptosis of SRA01/04 cells

**Figure 2.**
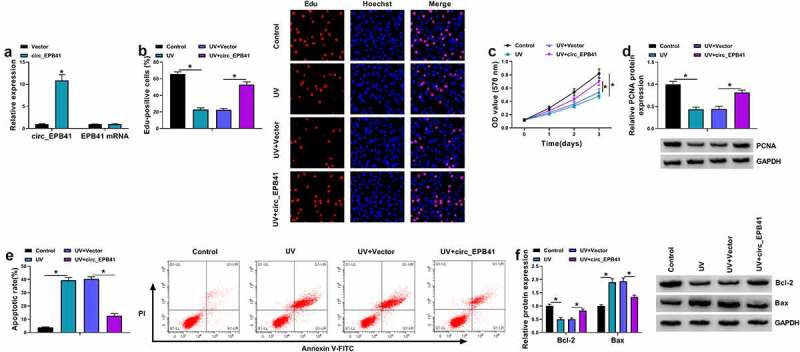
Enforced expression of circ_EPB41 attenuated the effects of UV irradiation on the proliferation and apoptosis of SRA01/04 cells. (a) The efficiency of circ_EPB41 overexpression was detected by qRT-PCR in SRA01/04 cells. (b and c) The effect of circ_EPB41 overexpression on UV irradiation-mediated cell proliferation was investigated by Edu and MTT assays in SRA01/04 cells. (d and f) The effects of UV irradiation and circ_EPB41 overexpression on the protein expression of PCNA, Bcl-2 and Bax were determined by western blot in SRA01/04 cells. (e) The effect of enforced expression of circ_EPB41 on UV irradiation-induced SRA01/04 cell apoptosis was confirmed by flow cytometry analysis. *P < 0.05

Whether circ_EPB41 was involved in the ARC occurrence was further analyzed. To validate this, SRA01/04 cells were treated with both UV irradiation and the plasmid overexpressing circ_EPB41. The data from Figure 2a showed that the transfection with the overexpression plasmid of circ_EPB41 led to a significant upregulation of circ_EPB41, but had no effect on EPB41 expression, which suggested the high efficiency of circ_EPB41 overexpression. Then, it was found that UV irradiation reduced the number of Edu-positive cells, OD values, and the protein expression of proliferation-related PCNA, whereas these effects were restored after circ_EPB41 overexpression (Figure 2b-d). In support, UV treatment induced G0/G1 phase cell arrest, which was attenuated after circ_EPB41 overexpression (Figure S1). Consistently, UV irradiation increased the apoptotic rate of SRA01/04 cells and the protein expression of apoptosis-related Bax, and decreased the expression of the apoptosis-linked protein Bcl-2; however, the effects induced by UV irradiation were relieved when circ_EPB41 expression was increased (Figure 2e and f). Furthermore, we treated SRA01/04 cells with the siRNA of circ_EPB41 to determine the consequential effect on cell apoptosis. The data from Figure S2A showed the success of circ_EPB41 knockdown. As presented in Figure S2B, circ_EPB41 silencing induced cell apoptosis. Thus, the above data demonstrated that circ_EPB41 assuaged UV irradiation-caused apoptosis of SRA01/04 cells.

## Circ_EPB41 acted as a sponge for miR-24-3p in SRA01/04 cells

**Figure 3.**
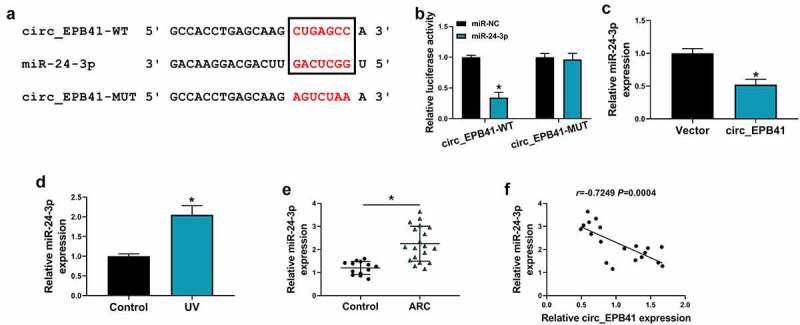
MiR-24-3p was associated with circ_EPB41. (a) The binding sites of circ_EPB41 for miR-24-3p and the mutant sites on circ_EPB41. (b) Dual-luciferase reporter assay was conducted to confirm the binding relationship of circ_EPB41 and miR-24-3p in SRA01/04 cells. (c) The effect of circ_EPB41 overexpression on miR-24-3p expression was determined by qRT-PCR in SRA01/04 cells. (d and e) MiR-24-3p expression was detected by qRT-PCR in SRA01/04 cells treated with UV irradiation, untreated SRA01/04 cells, anterior lens capsules of ARC (N = 19) and transparent anterior lens capsules (N = 13). (f) The association between circ_EPB41 and miR-24-3p expression in ARC tissues was determined by Spearman correlation analysis. *P < 0.05

The mechanism by which circ_EPB41 mediated UV irradiation-induced apoptosis of SRA01/04 cells was explored in this part. As displayed in Figure 3a, circ_EPB41 contained the complementary sites of miR-24-3p. Also, miR-24-3p could dramatically inhibit the luciferase activity of circ_EPB41-WT rather than that of circ_EPB41-MUT in SRA01/04 cells (Figure 3b). These findings demonstrated that circ_EPB41 bound to miR-24-3p. Then, the study found that miR-24-3p expression was downregulated in circ_EPB41-overexpressed SRA01/04 cells as compared with control (Figure 3c). Comparatively, miR-24-3p expression was upregulated in UV-irradiated SRA01/04 cells and anterior lens capsules of ARC (Figure 3d and e). In support, Spearman correlation analysis displayed the negatively linear correlation of miR-24-3p expression and circ_EPB41 expression in anterior lens capsules of ARC patients (Figure 3f).

### Circ_EPB41 ameliorated UV irradiation-induced apoptosis of SRA01/04 cells by interacting with miR-24-3p

**Figure 4.**
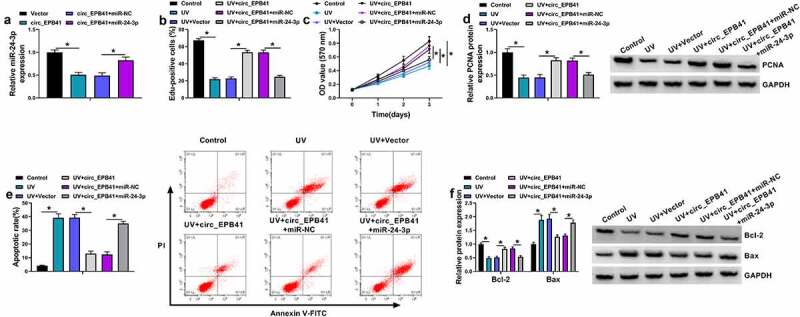
Circ_EPB41 regulated UV irradiation-induced apoptosis of SRA01/04 cells by interacting with miR-24-3p. (a) The effects between circ_EPB41 and miR-24-3p overexpression on miR-24-3p expression were determined by qRT-PCR in UV irradiation-stimulated SRA01/04 cells. (b-f) SRA01/04 cells were transfected with Vector, circ_EPB41, circ_EPB41+ miR-NC or circ_EPB41+ miR-24-3p after UV irradiation, with untreated SRA01/04 cells and UV irradiated-SRA01/04 cells as controls. (b and c) Cell proliferation was investigated by Edu and MTT assays. (d and f) The protein expression of PCNA, Bcl-2 and Bax was checked by western blot analysis. (e) Flow cytometry analysis was implemented to detect cell apoptosis. *P < 0.05

Given the binding relationship of circ_EPB41 and miR-24-3p, the study continued to explore whether miR-24-3p was involved in circ_EPB41-mediated apoptosis of SRA01/04 cells under UV irradiation. The data from the qRT-PCR analysis firstly showed that miR-24-3p overexpression attenuated the inhibitory effect of circ_EPB41 on miR-24-3p expression (Figure 4a). Then, we found that circ_EPB41 overexpression increased the number of Edu-positive cells, OD values, and PCNA protein expression after UV irradiation, whereas these effects were restored by miR-24-3p (Figure 4b-d). Comparatively, miR-24-3p mimics relieved the decreased cell apoptosis, reduced Bax protein expression, and increased Bcl-2 expression caused by circ_EPB41 overexpression after UV irradiation (Figure 4e and f). By the large, the above data demonstrated that miR-24-3p participated in the modulation of circ_EPB41 in UV irradiation-induced apoptosis of SRA01/04 cells.

### BPNT1 was identified as a target gene of miR-24-3p

**Figure 5.**
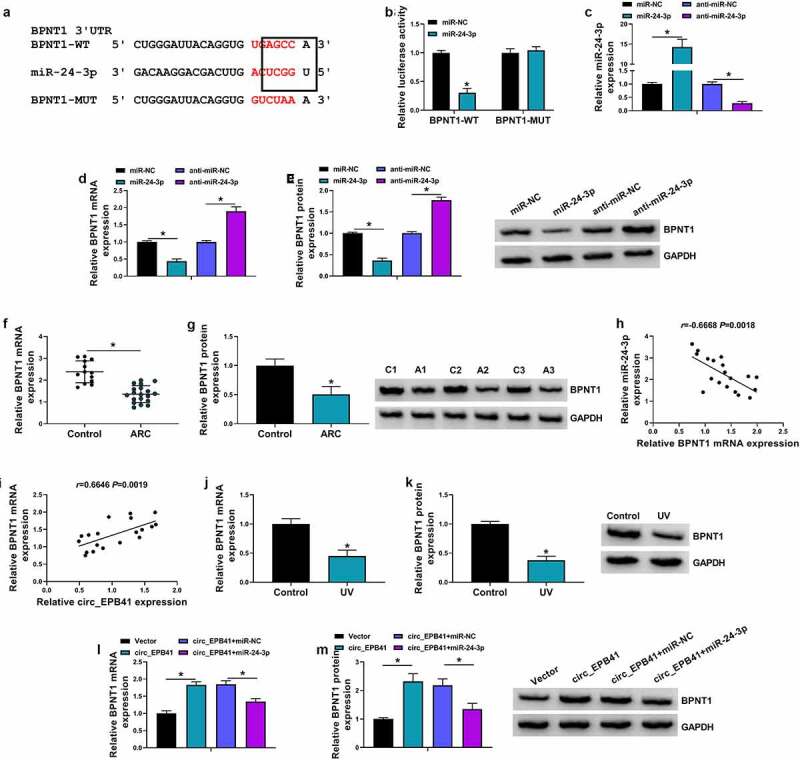
MiR-24-3p bound to BPNT1 in SRA01/04 cells. (a) The complementary sites of miR-24-3p with BPNT1 and the mutant sites on BPNT1. (b) Dual-luciferase reporter assay was used to identify the binding relationship of miR-24-3p and BPNT1 in SRA01/04 cells. (c) The efficiency of miR-24-3p overexpression and knockdown was determined by qRT-PCR in SRA01/04 cells. (d and e) The effects of miR-24-3p mimics and inhibitors on the expression of BPNT1 were demonstrated by qRT-PCR and western blot analysis in SRA01/04 cells. (f and g) The mRNA and protein expression of BPNT1 were detected by qRT-PCR and western blot, respectively, in anterior lens capsules of ARC and transparent anterior lens capsules. (h and i) Spearman correlation analysis was employed to assess the correlation of BPNT1 expression with miR-24-3p or circ_EPB41 expression in ARC tissues. (j and k) QRT-PCR and western blot were used to check BPNT1 expression in SRA01/04 cells treated with UV irradiation and untreated SRA01/04 cells. (l and m) The effects between circ_EPB41 and miR-24-3p on BPNT1 expression were determined by qRT-PCR and western blot analysis in SRA01/04 cells. *P < 0.05

To further explore the regulatory signaling pathway related to the effect of miR-24-3p on UV irradiation-induced cell apoptosis, we employed starbase online database for the prediction of the gene able to bind to miR-24-3p. We found that BPNT1 potentially bound to miR-24-3p (Figure 5a). The binding relationship between miR-24-3p and BPNT1 was identified by the dual-luciferase reporter assay (Figure 5b). For instance, exogenous miR-24-3p (mimic molecules) led to the inhibition of luciferase activity of BPNT1-WT rather than that of BPNT1-MUT. We then overexpressed or silenced miR-24-3p to determine their effects on the BPNT1 expression. qRT-PCR identified the efficiency of miR-24-3p overexpression or knockdown (Figure 5c). Subsequent data displayed that the mRNA and protein expression of BPNT1 were decreased by exogenous miR-24-3p, but increased by miR-24-3p inhibitors (Figure 5d and e). In addition, qRT-PCR and western blot were performed on ARC tissues to detect BPNT1 expression. As a result, the ARC tissues displayed a lower expression of BPNT1 as compared with controls (Figure 5f And G). Consistently, the study revealed that BPNT1 expression was negatively related to miR-24-3p, but positively to circ_EPB41 in ARC tissues (Figure 5h and i). As expected, BPNT1 was downregulated in UV-irradiated SRA01/04 cells compared with untreated SRA01/04 cells (Figure 5j and k). Furthermore, the data from Figure 5l and m showed that circ_EPB41 overexpression increased the mRNA and protein expression of BPNT1, whereas these effects were attenuated by miR-24-3p mimics, which indicated that circ_EPB41 could regulate BPNT1 expression by miR-24-3p.

### MiR-24-3p depletion assuaged UV irradiation-induced apoptosis of SRA01/04 cells by binding to BPNT1

**Figure 6.**
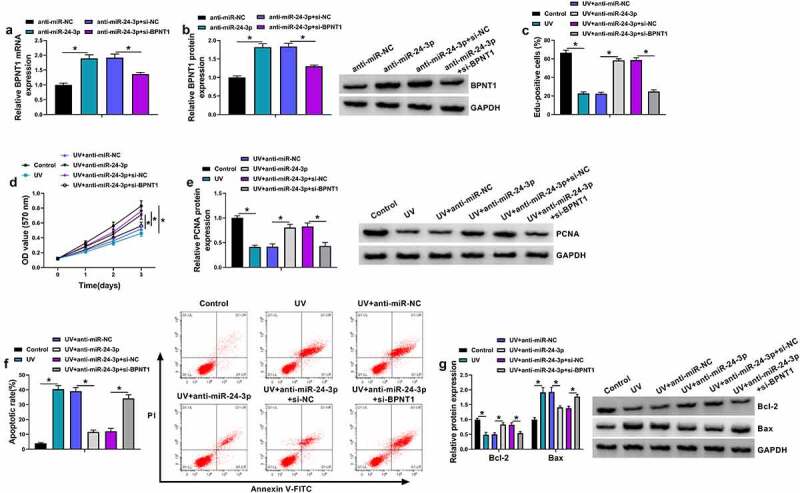
The effect of miR-24-3p depletion on UV irradiation-induced apoptosis was associated with BPNT1 in SRA01/04 cells. (a and b) The effects between miR-24-3p inhibitors and BPNT1 depletion on BPNT1 expression were determined by qRT-PCR and western blot analysis in SRA01/04 cells. (c-g) SRA01/04 cells were transfected with anti-miR-NC, anti-miR-24-3p, anti-miR-24-3p+si-NC or anti-miR-24-3p+si-BPNT1 after UV irradiation, with untreated SRA01/04 cells and UV irradiated-SRA01/04 cells as controls, and cell proliferation was determined by Edu and MTT assays (c and d), the protein expression of PCNA, Bcl-2 and Bax by western blot analysis (e and g), and cell apoptosis by flow cytometry analysis (f). *P < 0.05

We continued to explore whether miR-24-3p regulated UV irradiation-induced apoptosis of SRA01/04 cells through BPNT1. To demonstrate this, we silenced both miR-24-3p and BPNT1 to determine the consequential effects on the proliferation and apoptosis of SRA01/04 cells after UV irradiation. As shown in Figure 6a and B, the promoting effect of miR-24-3p inhibitors on BPNT1 expression was attenuated after BPNT1 knockdown. Subsequently, we found that miR-24-3p depletion promoted the proliferation of UV-irradiated SRA01/04 cells, accompanied by PCNA upregulation; however, these effects were attenuated by a reduced expression of BPNT1 (Figure 6c-e). A similar pattern of data among different groups was also found from flow cytometry analysis. For instance, miR-24-3p inhibitors reduced the apoptotic rate of UV-irradiated SRA01/04 cells, which was attenuated after BPNT1 knockdown (Figure 6f). In support, the increased expression of Bcl-2 and decreased expression of Bax caused by miR-24-3p downregulation were rescued after BPNT1 depletion in UV-irradiated SRA01/04 cells (Figure 6g). Thus, these evidences demonstrated that the effect of miR-24-3p on UV irradiation-induced apoptosis involved BPNT1 in SRA01/04 cells.

## Discussion

As the most common type of cataracts, ARC is the most major cause of loss of useful vision. In addition to aging, trauma and radiation exposure are also the factors leading to cataract [24]. At present, the exact pathogenesis of ARC has not been well elucidated, but previous evidences have suggested that the apoptosis of LECs contributes to the formation of ARC [2,25]. In addition, several researches have focused on the expression properties of circRNAs in ocular diseases, including ARC, and discovered that circRNAs have the potential as a therapeutic or diagnostic biomarker of cataracts [12,26]. On that account, we explored the role of circ_EPB41, a novel circRNA, in LECs induced by UV radiation. As a result, we found that circ_EPB41 overexpression ameliorated UV radiation-caused apoptosis of LECs, and elucidated that the behind mechanism was related to miR-24-3p and BPNT1.

As a newly rediscovered noncoding RNA, circRNA has become a research hotspot in disclosing the precise molecular mechanism of different types of diseases. In recent years, some evidences suggested that circRNA served vital parts in the pathogenesis of ocular diseases [27]. In a study conducted by Liu *et al*., we found the decreased expression of circRNA homeodomain interacting protein kinase 3 (circHIPK3) in cortical, nuclear as well as posterior subcapsular ARC, and the depletion of the circRNA inhibited the biological behaviors of LECs, like cell growth and anti-apoptosis [12]. CircRNA zinc finger protein 292 (circZNF292) was capable of attenuating hydrogen peroxide-caused promotion of cell apoptosis and oxidative stress, and the underneath mechanism was manipulated by circZNF292-miR-222-3p-E2F transcription factor 3 (E2F3) regulatory axis [28]. Besides, Liu and his colleagues indicated that circular RNA circMRE11A_013 (circMRE11A) knockdown contributed to the proliferation of SRA01/04 cells by initiating ataxia-telangiectasia mutated kinase (ATM)/p53/p21 signaling pathway through interaction with UBX domain-containing protein 1 [29]. In the present research, we revealed the function of circ_EPB41 in ARC formation for the first time. Herein, the anterior lens capsules of ARC displayed the lower expression of circ_EPB41 as compared to transparent anterior lens capsules. Comparative data also confirmed that the circRNA expression was reduced in UV-radiated LECs. Subsequent data elucidated that circ_EPB41 was chiefly located in the cytoplasm of LECs. Besides, it was discovered that UV radiation-induced proliferation inhibition and apoptosis promotion were attenuated after circ_EPB41 overexpression in LECs.

MiRNA is a conserved short RNA (18–25 nucleotides) that has been found to mediate the biological processes of various types of cells, such as cell proliferation, apoptosis, and differentiation [30]. Considerable data showed that the pathogenesis of cataract was related to the altered expression of miRNAs [31]. In particular, miRNA participated in regulating the function of LECs [32]. Some investigators have explored the function of miRNA during cataract formation [33]. In this work, miR-24-3p was identified as the target miRNA of circ_EPB41. Cross-sectional report elucidated the important role of miR-24-3p in age-related macular degeneration pathology [34]. Also, miR-24-3p introduction promoted LEC death under oxidative stress condition by interacting with p53 [35]. Here, miR-24-3p was negatively regulated by circ_EPB41, and was highly expressed in anterior lens capsules caused by ARC and UV-radiated LECs in comparison with controls. Also, we provided the evidences that miR-24-3p inhibitors assuaged the effects of UV radiation on LEC apoptosis and proliferation. Importantly, the study demonstrated that circ_EPB41 regulated UV radiation-caused cell apoptosis by binding to miR-24-3p.

MiRNA commonly regulates gene expression by pairing it to its 3ʹ-untranslated region. In the report, we discovered that miR-24-3p was able to bind to BPNT1. BPNT1, also called as BPntase, is located in cytoplasm and belongs to magnesium-dependent phosphomonoesterase family [36]. Functional studies indicated that BPNT1 was involved in sulfation-independent processes, such as methionine biosynthesis [37]. Some investigators predicted the downregulation of BPNT1 in heat-shock transcription factor 4 (HSF4)-null lens compared with wild-type lens through miRNA-DEG regulatory network [38]. However, there was no data involving the role of BPNT1 in UV radiation-caused ARC. The study was the first one to report the function of BPNT1 in ARC formation. Herein, we found BPNT1 was weakly expressed in ARC tissues and UV-radiated LECs in relative to controls. MiR-24-3p-overexpressed LECs displayed a low level of BPNT1, whereas miR-24-3p-knockdowned cells had the opposite effect. Additionally, the study demonstrated that the repressing effects of miR-24-3p inhibitors on UV radiation-caused proliferation repression and apoptosis promotion were remitted after BPNT1 depletion, which indicated that miR-24-3p regulated UV radiation-induced LEC injury through BPNT1. Furthermore, our data elucidated that circ_EPB41 induced BPNT1 expression through miR-24-3p in LECs.

However, two shortcomings needed to be considered when evaluating these findings. Firstly, the present study lacked the direct evidence that circ_EPB41/miR-24-3p/BPNT1 axis regulated UV radiation-induced LEC injury. Additionally, the new mechanism was only testified in cell model assay, and mouse model experiment involving the regulatory role and mechanism of circ_EPB41 in UV-caused ARC were absent. The above two issues would be addressed in subsequent study.

## Conclusion

**Figure 7.**
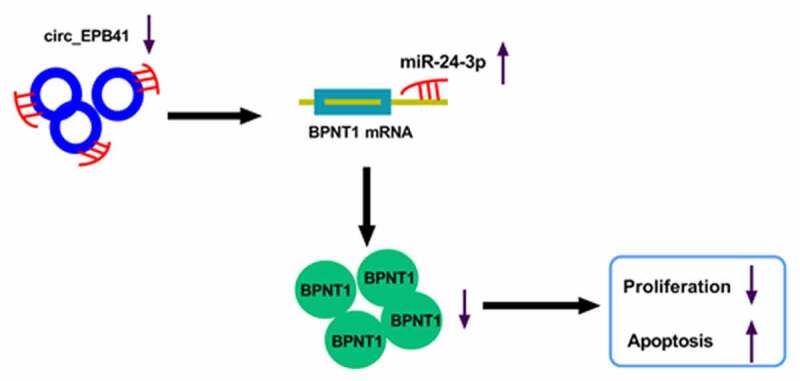
The schematic diagram of the underneath mechanism responsible for the function of circ_EPB41 in UV irradiation-induced apoptosis of LECs

Taken together, UV radiation led to the repression of circ_EPB41 expression, and the altered expression of the circRNA reduced the BPNT1 level by binding to miR-24-3p, thereby inhibiting the proliferation and inducing apoptosis of LECs (Figure 7). The novel mechanism provides us with a reliable target for ARC therapy.

## Supplementary Material

Supplemental Material
