# Supplementary material for: Circular RNA erythrocyte membrane protein band 4.1 assuages ultraviolet irradiation-induced apoptosis of lens epithelial cells by stimulating 5’-bisphosphate nucleotidase 1 in a miR-24-3p-dependent manner
Source: Bioengineered. 2021 Oct 28;12(1):8953–64. doi: 10.1080/21655979.2021.1990196 (PMC8806953; doi:10.1080/21655979.2021.1990196)
Supplement: Supplemental Material [file KBIE_A_1990196_SM9737.zip › supplementary figure captions.docx]

**Figure S1** **The effects between UV treatment and circ_EPB41 overexpression on SRA01/04 cell cycle were determined by DNA content quantitation assay.** **P*<0.05.

**Figure S2 The effect of circ_EPB41 knockdown on SRA01/04 cell apoptosis.** (A) The efficiency of circ_EPB41 knockdown was determined by qRT-PCR in SRA01/04 cells. (B) Cell apoptosis was analyzed by flow cytometry analysis in the SRA01/04 cells treated with si-NC or si-circ_EPB41. **P*<0.05.
