# Supplementary figures and images for: Circular RNA erythrocyte membrane protein band 4.1 assuages ultraviolet irradiation-induced apoptosis of lens epithelial cells by stimulating 5’-bisphosphate nucleotidase 1 in a miR-24-3p-dependent manner
Source: Bioengineered. 2021 Oct 28;12(1):8953–64. doi: 10.1080/21655979.2021.1990196 (PMC8806953; doi:10.1080/21655979.2021.1990196)

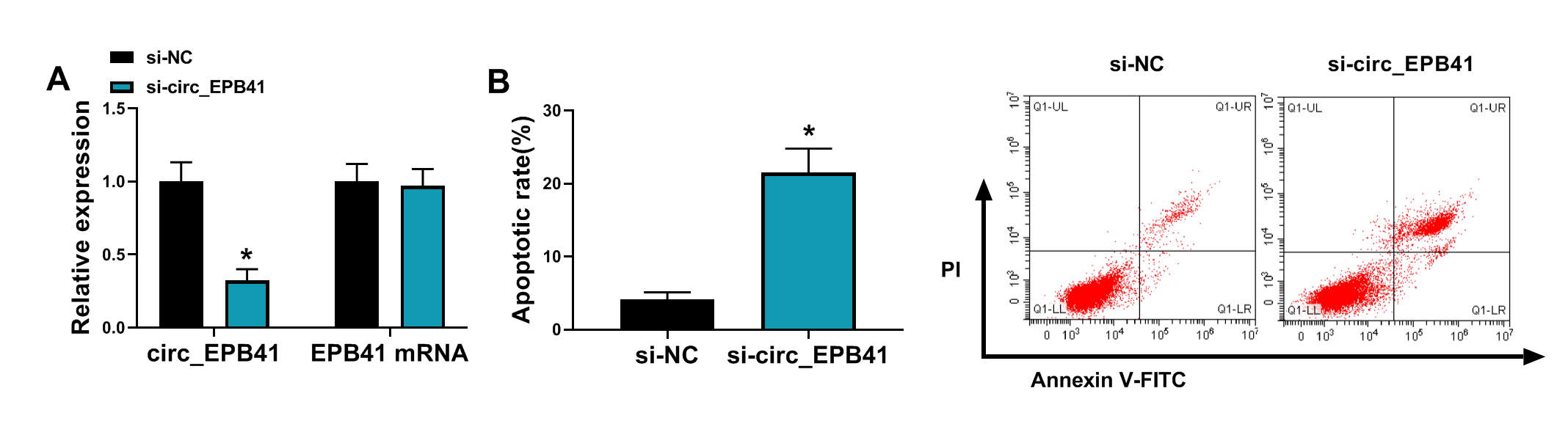

Supplement: Supplemental Material [file KBIE_A_1990196_SM9737.zip › Fig S1 revised.tif]

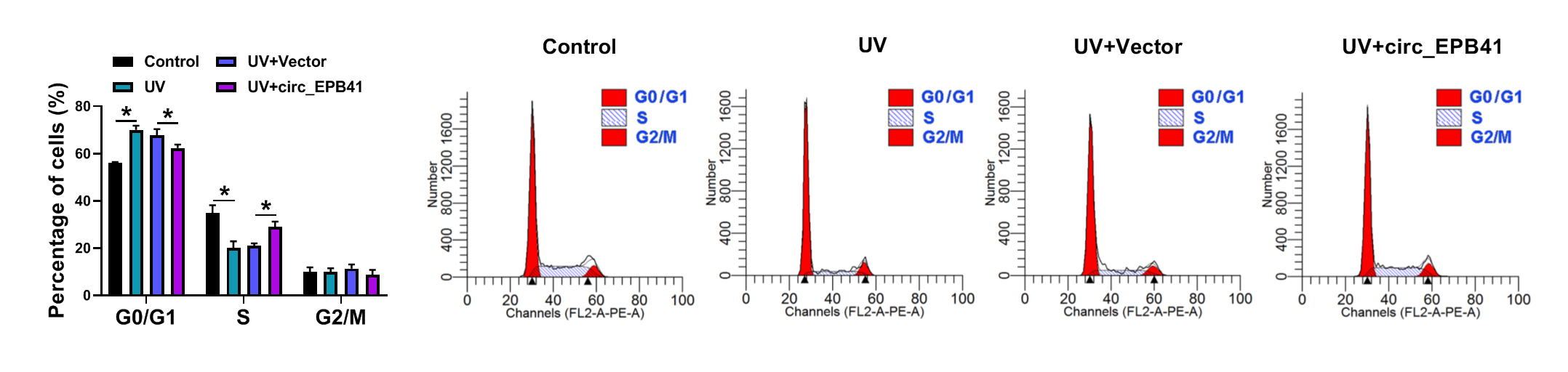

Supplement: Supplemental Material [file KBIE_A_1990196_SM9737.zip › Fig S2 revised.tif]
